# Synthesis, and Antitumor Activity of Some *N1*-(Coumarin-7-yl) Amidrazones and Related Congeners

**DOI:** 10.3390/molecules16054305

**Published:** 2011-05-24

**Authors:** Mohammad S. Mustafa, Mustafa M. El-Abadelah, Malek A. Zihlif, Randa G. Naffa, Mohammad S. Mubarak

**Affiliations:** 1 Department of Chemistry, The University of Jordan, Amman 11942, Jordan; 2 Department of Pharmacology, Faculty of Medicine, The University of Jordan, Amman 11942, Jordan; 3 Molecular Biology Research Laboratory, Faculty of Medicine, The University of Jordan, Amman 11942, Jordan

**Keywords:** 7-aminocoumarins, *N*-(coumarin-7-yl)hydrazonoyl chloride, 1-piperazinyl amidrazones, nitrileimine, antitumor activity

## Abstract

A series of new *N*1-(coumarin-7-yl)amidrazones incorporating *N*-piperazines and related congeners were synthesized by reacting the hydrazonoyl chloride derived from 7-amino-4-methylcoumarin with the appropriate piperazines. The chemical structures of the newly prepared compounds were supported by elemental analyses, ^1^H-NMR, ^13^C-NMR, and ESI-HRMS spectral data. The antitumor activity of the newly synthesized compounds was evaluated. Among all the compounds tested, 7-{2-[1-(4-(1-benzyl-2-ethyl-4-nitro-1H-imidazol-5-yl)piperazin-1-yl)-2-oxopropylidene]hydrazinyl}-4-methyl-2H-chromen-2-one (**3n**) was the most potent against MCF-7 and K562 cells, with IC_50_ values of 20.2 and 9.3 μM, respectively.

## 1. Introduction

Due to their structural and therapeutic diversity as pharmaceutical agents, along with their commercial availability, piperazine derivatives continue to capture the attention of synthetic and medicinal chemists. Piperazine-based compounds have been employed as antibacterial, antidepressant, and antitumor drugs, and as α-adrenoceptor antagonists, CCR5 receptor antagonists, 5-HT7 receptor antagonists, and adenosine A2a receptor antagonists [1]. Several piperazine derivatives have reached the stage of clinical application; among the known drugs that are used to treat anxiety is a pyrimidinyl piperazinyl compound (buspirone, BuSpar^®^) [2], while a 3-chlorophenyl piperazinyl drug (trazodone, Desyrel^®^) is used as an antidepressant [3]. Besides, several publications have dealt with the synthesis and evaluation of thrombin inhibitors that incorporate an amidrazone functionality as a structural motif [4,5,6], and there is a report pertaining to the inactivation of lipoxygenase-1 from soybeans by open-chain and cyclic amidrazones [7].

On the other hand, coumarin derivatives have drawn considerable attention from researchers due to their role in natural and synthetic organic chemistry, and their interesting biological activities. Compounds which contain a coumarin nucleus were found to exhibit various biological activities such as anticoagulant and antithrombotic properties [8]. Some derivatives have shown molluscicidal, anthelmintic [9], hypnotic, and insecticidal [10] activity, while others have served as antifungal [11], anti-inflammatory [12] and antiviral agents, including against human immunodeficiency virus [13], and anticoagulant properties [14]. In addition, coumarins have also been used as additives in food and cosmetics [15], and in the preparation of optical brighteners, dispersed fluorescent and laser dyes [16].

In view of the widespread interest in the activity spectrum and profile of coumarins [17,18,19,20], and in continuation of our work on the synthesis of new compounds of pharmacological and biological interest [1,21,22,23,24], we describe herein the preparation and spectroscopic characterization of some new piperazinyl amidrazones containing coumarin moieties and evaluation of their antitumor activity.

## 2. Results and Discussion

### 2.1. Chemistry

**Scheme 1:**
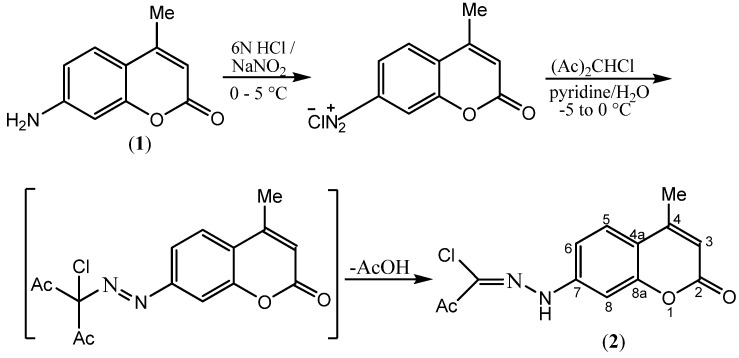
Synthesis of *N*-(4-methyl-2-oxo-2H-chromen-7-yl)-2-oxopropanehydrazonoyl chloride (**2**).

The hydrazonoyl chloride synthon **2** required in this study is prepared *via* direct coupling of 4-methylcoumarin-7-diazonium chloride with 3-chloropentane-2,4-dione in aqueous-ethanolic sodium acetate (Japp-Klingemann reaction) [25,26,27] (Scheme 1). An acidic solution of the former coumarin-7-diazonium chloride is freshly prepared by diazotization of 7-amino-4-methylcoumarin (suspended in 6N aq. HCl) which, in turn, is prepared from *m*-aminophenol according to a reported procedure [28,29].

**Scheme 2:**
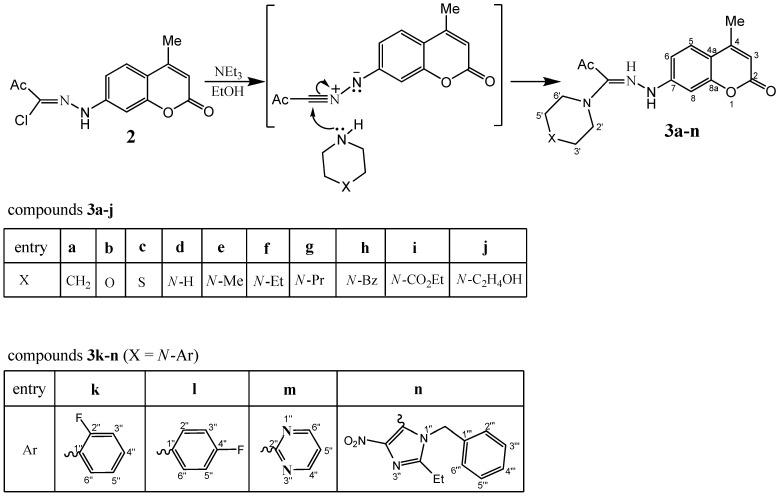
Synthesis of 4-methyl-7-{2-[2-oxo-1-(substituted N-hexahydroazinyl)propylidene]-hydrazinyl}-2*H*-chromen-2-ones **3a-n**.

Piperazine, *N*-substituted piperazines and related cyclic secondary amine congeners, acting as nitrogen nucleophiles, are expected to add readily to *N*-(4-methylcoumarin-7-yl)nitrile imine (the reactive 1,3-dipolar species generated *in situ* from the corresponding hydrazonoyl chloride precursor **2** in the presence of triethylamine) to give the respective amidrazone adducts **3a-n** (Scheme 2). This mode of nucleophilic addition reaction of various nucleophiles to 1,3-dipoles is well-documented [30,31,32,33,34,35,36,37,38] and several adducts related to **3** were obtained from the reaction of amines with hydrazonoyl chlorides.

The newly synthesized compounds **3a-n **were characterized by elemental analyses, MS and NMR spectral data. These data, detailed in the experimental part, are consistent with the suggested structures. Thus, the mass spectra display the correct molecular ion peaks for which the measured high resolution (HRMS) data are in good agreement with the calculated values. DEPT and 2D (COSY, HMQC, HMBC) experiments showed correlations that helped in the ^1^H- and ^13^C-signal assignments to the different carbons and their attached, and/or neighboring hydrogens.

### 2.2. Antitumor Activity

The antitumor activities of compounds **3c-n** were characterized by conducting cell viability assays using tetrazolium dye 3-(4,5-dimethylthiazol-2-yl)-2,5-diphenyltetrazolium bromide (MTT). Cultures of MCF7 breast cancer cells and K562 human leukemia cells were treated first at one concentration of 50 μg/mL and the results are shown in Table 1.

**Table 1:** Percentage cell survival of MCF-7 and K562 cells following 72 hours exposure to 50 μM f all compounds.

Compound	MCF-7 % survival ± standard deviation	K562 % survival ± standard deviation
**3a**	81.8 ± 4.8	84.1 ± 3.8
**3b**	81.7 ± 10.6	82.3 ± 4.4
**3c**	89.8 ± 13.7	94.3 ± 6.3
**3d**	86.4 ± 5.8	72.7 ± 1.5
**3e**	89.7 ± 8.9	85.3 ± 3.0
**3f**	91.4 ± 5.9	90.7 ± 2.3
**3g**	81.5 ± 4.7	91.1 ± 3.5
**3h**	80.6 ± 1.6	89.6 ± 3.1
**3i**	46.8 ± 3.5	92.6 ± 12.2
**3j**	85. 6 ± 2.8	86.5 ± 3.6
**3k**	85.9 ± 6.0	96.4 ± 12.4
**3l**	84.6 ± 11.0	93.4 ± 2.2
**3m**	36.2 ± 5.7	101.3 ± 3.0
**3n**	43. 3 ± 5.1	52.7 ± 6.1

Compounds **3i**, **3m** and **3n** showed potential anti-MCF-7activity. These compounds were able to reduce the viability after 72 hours to less than 50%. With respect to K562 cells, only compound **3n** showed considerable inhibition of cell proliferation. Furthermore, we explored the anti-tumor activity for **3i**, **3m** and **3n** (compounds that showed potential activities) in two more cancer cells: breast cancer cell line ZR-75-1 and leukemia cell line HL60. We determined the IC_50_ values for compounds **3i**, **3m** and **3n** on ZR-75-1 and HL60 additional cell lines, and on the MCF-7 and K526 cell lines as well (Table 2). Notably, compound **3n** was the most potent against MCF-7 cells scoring an IC_50_ value of 20.2 μM and showing a very promising activity against the K562 cells with an IC_50_ of 9.3 μM (Table 2).

**Table 2:** Effects of compounds **3i,** **3m** and **3n** on MCF-7, ZR-75-1, K562 and HL60.

Compound	IC_50_ MCF-7 (μM)	IC_50_ ZR-75-1 (μM)	IC_50_ K562 (μM)	IC_50_ HL60 (μM)
**Doxorubicin**	0.31 ± 0.01	1.0 ± 0.01	3.54 ± 0.54	0.09 ± 0.005
**3i**	47.8 ± 3.3	>100	>100	>100
**3m**	39.7 ± 1.7	>100	>100	>100
**3n**	20.2 ± 3.7	>100	9.2 ± 2.8	>100

From the structure-activity relationships point of view, the nature of substituents on the X position seems to play a critical role for the cytotoxic activity. For example, in case of MCF-7 breast cancer cells, compounds **3i**, **3m**, and **3n** with the substituent N-CO_2_Et, N-(2-pyrimidyl), and *N*-(1-benzyl-2-ethyl-4-nitroimidazol-5-yl) appendages, respectively, exhibited cytotoxic IC_50_ values of 49, 38, and 20 μM. The shared characteristic feature between these three substituents, which are absent in all others, is the presence of the nucleophilic component next to position X. However, this belief cannot be generalized on the in anti-K562 cell activity, where compound **3n** was the only active compound and no activity could be observed with compounds **3i** and **3m**. What make compound **3n** active against K562 cells, giving a relatively low IC_50 _of 9 μM, may be linked with the bulky substituent that characterized this compound from all others tested. This bulky substituent on compound **3n** may also be associated with the lowest IC_50_ value as exhibited by this compound against the MCF-7 cells.

## 3. Experimental

### 3.1. General

The following chemicals, used in this study, were purchased from Acros and were used as received: piperidine, morpholine, thiomorpholine, piperazine, *N*-alkylpiperazines, *N*-arylpiperazines, ethyl *N*-piperazinecarboxylate. 1-(1-Benzyl-2-ethyl-4-nitro-1*H*-imidazol-5-yl)piperazine was prepared according to a literature procedure [39]. Silica-gel for column chromatography was purchased from Macherey-Nagel GmbH & Co (Germany). Melting points (uncorrected) were determined on a Stuart scientific melting point apparatus in open capillary tubes. ^1^H- and ^13^C-NMR spectra were recorded on a 300 MHz spectrometer (Bruker DPX-300) with *TMS* as the internal standard. Chemical shifts are expressed in δ units; *J*-values for ^1^H-^1^H, ^1^H-F and ^13^C-F coupling constants are given in Hertz. High resolution mass spectra (HRMS) were acquired (in positive or negative mode) using electrospray ion trap (ESI) technique by collision-induced dissociation on a Bruker APEX-4 (7-Tesla) instrument. The samples were dissolved in acetonitrile, diluted in spray solution (methanol/water 1:1 v/v + 0.1% formic acid) and infused using a syringe pump with a flow rate of 2 µL/min. External calibration was conducted using arginine cluster in a mass range *m/z* 175-871. 

### 3.2. 7-Amino-4-methylcoumarin (***1***)

This synthon, required in the present study, was prepared according to a literature procedure [28,29] which involves reaction of *m*-aminophenol with methoxycarbonyl chloride as the initial step; the resulting *N*-protected *m*-aminophenol underwent cyclocondensation upon reaction with ethyl acetoacetate and conc. sulpuric acid, followed by removal of the *N*-protecting group (*via* treatment with sodium hydroxide) to deliver the title compound **1**; mp = 224-226 °C (lit. mp = 226-227 °C) [28,29].

### 3.3. N-(4-Methyl-2-oxo-2H-chromen-7-yl)-2-oxo-propanehydrazonoyl chloride (***2***)

Compound **1** (17.5 g, 0.10 mol) was dissolved in 17% aqueous hydrochloric acid (160 mL). To this solution was added drop-wise a solution of sodium nitrite (7.6 g, 0.11 mol) in water (15 mL) with efficient stirring at 0-5 °C. Stirring was continued for 20-30 min., and the resulting fresh cold 4-methyl-2-oxo-2*H*-chromene-7-diazonium chloride [also named as 7-(chlorodiazenyl)-4-methyl-coumarin] solution was poured onto cold solution (0 to -10 °C, ice-salt bath) of 3-chloropentan-2,4-dione (13.5 g, 0.1 mol) in ethanol/water (160 mL, 1:1 v/v) containing 30.0 g of sodium acetate with vigorous stirring. The resulting yellowish-colored mixture was further stirred until a solid precipitate was formed (5-10 min). The reaction mixture was then diluted with cold water (200 mL), the solid product was collected by suction filtration, washed several times with cold water, dried, and recrystallized from acetonitrile. Yield 25.4 g (91%); Mp = 271-274 °C. ^1^H-NMR (300 MHz, DMSO-d_6_): δ = 2.34 (d, *J* = 1.0 Hz, 3H, C*H*_3_-4), 2.46 (s, 3H, O=C-C*H*_3_), 6.17 (d, *J* =1.0 Hz, 1H, H-3), 7.27 (d, *J* = 1.8 Hz, 1H, H-8), 7.38 (dd, *J* = 8.7, 1.8 Hz, 1H, H-6), 7.67 (d, *J* = 8.7 Hz, 1H, H-5), 10.97 (s, 1H, N-H). ^13^C-NMR (75 MHz, DMSO-d_6_): δ = 18.6 (*C*H_3_-4), 26.0 (O=C-*C*H_3_), 101.9 (C-8), 111. 8 (C-6), 112.1 (C-3), 115.1 (C-4a), 127.1 (C-5), 146.0 (C-4), 146.2 (C-7), 153.8 (-C=N), 154.9 (C-8a), 160.5 (C-2), 188.6 (O=*C*-Me). Anal. Calcd. for C_13_H_11_ClN_2_O_3_ (278.69 g/mol): C, 56.03; H, 3.98; Cl, 12.72; N, 10.05. Found: C, 56.18; H, 4.05; Cl, 12.56; N, 9.93. ESI-HRMS *m/z*: Calcd. for C_13_H_12_ClN_2_O_3_ [M + H]^+^ 279.05364. Found: 279.05310.

### 3.4. General procedure for the preparation of 4-Methyl-7-{2-[2-oxo-1-(substituted N-hexahydro-azinyl)propylidene]hydrazinyl}-2H-chromen-2-ones ***3a-n***

To a cold suspension (0 to -10 °C) of compound **2 **(0.5 g, 1.8 mmol) in ethanol (20 mL) was added, with stirring, a solution of the appropriate secondary amine (2.0 mmol) and triethylamine (3 mL) in ethanol (10 mL). Stirring was continued at 0-5 °C for 2-4 h, and then at ambient temperature for 10-12 h. The solvent was then removed under reduce pressure and the residue was treated with water (15 mL). The resulting crude solid product was collected by suction filtration, washed with water, dried and purified on preparative silica gel TLC plates.

*4-Methyl-7-{2-[2-oxo-1-(piperidin-1-yl)propylidene]hydrazinyl}-2H-chromen-2-one* (**3a**): Yield = 0.45 g (76.8%); Mp = 190-192 °C. ^1^H-NMR (300 MHz, DMSO-d_6_): δ = 1.50 (m, 2H, H_2_-4'), 1.63 (m, 4H, H_2_-3' + H_2_-5'), 2.34 (d, *J* = 1.0 Hz, 3H, C*H*_3_-4), 2.35 (s, 3H, O=C-C*H*_3_), 2.89 (m, 4H, H_2_-2' + H_2_-6' ), 6.11 (d, *J* = 1.0 Hz, 1H, H-3), 7.25 (d, *J* = 2.0 Hz, 1H, H-8), 7.34 (dd, *J* = 8.7, 2.0 Hz, 1H, H-6), 7.65 (d, *J* = 8.7 Hz, 1H, H-5), 9.83 (s, 1H, N-H). ^13^C-NMR (75 MHz, DMSO-d_6_): δ = 18.5 (CH_3_-4), 24.4 (C-4'), 25.7 (C-3'/C-5'), 26.6 (O=C-*C*H_3_), 49.1 (C-2'/C-6'), 100.9 (C-8), 111.6 (C-6), 111.1 (C-3), 113.8 (C-4a), 126.8 (C-5), 146.0 (C-4), 147.4 (C-7), 153.9 (-C=N), 155.1 (C-8a), 160.7 (C-2), 195.5 (O=*C*-Me). Anal. Calcd. for C_18_H_21_N_3_O_3 _(327.38 g/mol): C, 66.04; H, 6.47; N, 12.84. Found: C, 65.88; H, 6.41; N, 12.72. ESI-HRMS *m/z*: Calcd. for C_18_H_20_N_3_O_3 _[M − H]^-^ 326.15047. Found: 326.14992.

*4-Methyl-7-{2-[1-morpholino-2-oxopropylidene]hydrazinyl}-2H-chromen-2-one* (**3b**): Yield = 0.41 g (69.2%); Mp = 213-215 °C. ^1^H-NMR (300 MHz, CDCl_3_): δ = 2.39 (d, *J* = 1.0 Hz, 3H, C*H*_3_-4), 2.42 (s, 3H, O=C-C*H*_3_), 3.07 (m, 4H, H_2_-2' + H_2_-6' ), 3.80 (m, 4H, H_2_-3' + H_2_-5' ), 6.12 (d, *J* = 1.0 Hz, 1H, H-3), 7.06 (dd, *J* = 8.7, 2.0 Hz, 1H, H-6), 7.19 (d, *J* = 2.0 Hz, 1H, H-8), 7.51 (d, *J* = 8.7 Hz, 1H, H-5), 9.32 (s, 1H, N-H). ^13^C-NMR (75 MHz, CDCl_3_): δ = 18.5 (CH_3_-4), 26.6 (O=C-*C*H_3_), 66.5 (C-3'/C-5'), 48.2 (C-2'/C-6'), 101.2 (C-8), 111.3 (C-6), 111.7 (C-3), 113.9 (C-4a), 126.8 (C-5), 144.3 (C-4), 147.3 (C-7), 153.8 (-C=N), 155.1 (C-8a), 160.7 (C-2), 195.3 (O=*C*-Me). Anal. Calcd for C_17_H_19_N_3_O_4_ (329.35 g/mol): C, 62.00; H, 5.81; N, 12.76. Found: C, 62.14; H, 5.78; N, 12.65. ESI-HRMS *m/z*: Calcd for C_17_H_18_N_3_O_4 _[M − H]^-^ 328.12973. Found: 328.12925.

*4-Methyl-7-{2-[2-oxo-1-thiomorpholinopropylidene]hydrazinyl}-2H-chromen-2-one* (**3c**)*:* Yield = 0.45 g (72.4%); Mp = 236-238 °C. ^1^H-NMR (300 MHz, CDCl_3_): δ = 2.39 (d, *J* = 1.0 Hz, 3H, C*H*_3_-4), 2.41 (s, 3H, O=C-C*H*_3_), 2.75 (m, 4H, H_2_-3' + H_2_-5'), 3.26 (m, 4H, H_2_-2' + H_2_-6'), 6.14 (d, *J* = 1.0 Hz, 1H, H-3), 7.03 (dd, *J* = 8.7, 2.0 Hz, 1H, H-6), 7.18 (d, *J* = 2.0 Hz, 1H, H-8), 7.52 (d, *J* = 8.7 Hz, 1H, H-5), 9.12 (s, 1H, N-H). ^13^C-NMR (75 MHz, CDCl_3_): δ = 18.7 (*C*H_3_-4), 25.9 (O=C-*C*H_3_), 28.5 (C-3'/C-5'), 50.3 (C-2'/C-6'), 101.6 (C-8), 110.7 (C-6), 112.3 (C-3), 114.7 (C-4a), 125.9 (C-5), 145.4 (C-4), 145.6 (C-7), 152.5 (-C=N), 155.3 (C-8a), 161.2 (C-2), 195.0 (O=*C*-Me). Anal. Calcd. for C_17_H_19_N_3_O_3_S (345.42 g/mol): C, 59.11; H, 5.54; N, 12.17. Found: C, 58.92; H, 5.51; N, 12.06. ESI-HRMS *m/z*: Calcd. for C_17_H_19_N_3_NaO_3_S [M + Na]^+^ 368.10448. Found: 368.10393.

*4-Methyl-7-{2-[2-oxo-1-(piperazin-1-yl)propylidene]hydrazinyl}-2H-chromen-2-one* (**3d**): Yield = 0.36 g (61%); Mp = 219-222 °C. ^1^H-NMR (300 MHz, CDCl_3_): δ = 1.65 (s, 1H, N(4')-H), 2.39 (d, *J* = 1.0 Hz, 3H, C*H*_3_-4), 2.41 (s, 3H, O=C-C*H*_3_), 2.98 (m, 4H, H_2_-2' + H_2_-6' ), 2.99 (m, 4H, H_2_-3' + H_2_-5' ), 6.13 (d, *J* = 1.0 Hz, 1H, H-3), 7.04 (dd, *J* = 8.7, 2.0 Hz, 1H, H-6), 7.16 (d, *J* = 2.0 Hz, 1H, H-8), 7.51 (d, *J* = 8.7 Hz, 1H, H-5), 9.29 (s, 1H, N-H). ^13^C-NMR (75 MHz, CDCl_3_): δ = 18.7 (*C*H_3_-4), 26.0 (O=C-*C*H_3_), 46.6 (C-3'/C-5'), 49.3 (C-2'/C-6'), 101.5 (C-8), 110.7 (C-6), 112.1 (C-3), 114.5 (C-4a), 125.9 (C-5), 145.0 (C-4), 145.7 (C-7), 152.5 (-C=N), 155.3 (C-8a), 161.3 (C-2), 195.2 (O=*C*-Me). Anal. Calcd. for C_17_H_20_N_4_O_3_ (328.37 g/mol): C, 62.18; H, 6.14; N, 17.06. Found: C, 62.04; H, 6.08; N, 16.92. ESI-HRMS *m/z*: Calcd. for C_17_H_21_N_4_O_3 _[M + H]^+^ 329.16137. Found: 329.16083.

*4-Methyl-7-{2-[1-(4-methylpiperazin-1-yl)-2-oxo-propylidene]hydrazinyl}-2H-chromen-2-one* (**3e**): Yield = 0.50 g (81.2%); Mp = 182-183 °C. ^1^H-NMR (300 MHz, CDCl_3_): δ = 2.36 (d, *J* = 1.0 Hz, 3H, C*H*_3_-4), 2.40 (s, 3H, O=C-C*H*_3_), 2.43 (s, 3H, N-C*H*_3_), 2.63 (m, 4H, H_2_-3' + H_2_-5' ), 3.14 (m, 4H, H_2_-2' + H_2_-6' ), 6.12 (d, *J* = 1.0 Hz, 1H, H-3), 7.07 (dd, *J* = 8.7, 2.0 Hz, 1H, H-6), 7.16 (d, *J* = 2.0 Hz, 1H, H-8), 7.52 (d, *J* = 8.7 Hz, 1H, H-5), 9.23 (s, 1H, N-H). ^13^C-NMR (75 MHz, CDCl_3_): δ = 18.7 (C-4), 25.9 (O=C-*C*H_3_), 46.4 (N-*C*H_3_), 47.9 (C-2'/C-6'), 55.7 (C-3'/C-5'), 101.4 (C-8), 110.7 (C-6), 112.0 (C-3), 114.4 (C-4a), 125.8 (C-5), 145.0 (C-4), 145.8 (C-7), 152.5 (-C=N), 155.2 (C-8a), 161.2 (C-2), 195.0 (O=*C*-Me). Anal. Calcd. for C_18_H_22_N_4_O_3_ (342.39 g/mol): C, 63.14; H, 6.48; N, 16.36. Found: C, 63.22; H, 6.51; N, 16.23. ESI-HRMS *m/z*: Calcd. for C_18_H_23_N_4_O_3 _[M + H]^+^ 343.17702. Found: 343.17652.

*7-{2-[1-(4-Ethylpiperazin-1-yl)-2-oxopropylidene]hydrazinyl}4-methyl-2H-chromen-2-one* (**3f**)*:* Yield = 0.40 g (62.4%); Mp = 165-168 °C. ^1^H-NMR (300 MHz, CDCl_3_): δ = 1.12 (t, *J* = 7.2 Hz, 3H, C*H*_3_-CH_2_-), 2.50 (q, 2H, *J* = 7.2 Hz, CH_3_-C*H*_2_-N), 2.39 (d, *J* = 1.0 Hz, 3H, C*H*_3_-4), 2.42 (s, 3H, O=C-C*H*_3_), 2.58 (m, 4H, H_2_-3' + H_2_-5' ), 3.11 (m, 4H, H_2_-2' + H_2_-6' ), 6.12 (d, *J* = 1.0 Hz, 1H, H-3), 7.05 (dd, *J* = 8.7 Hz, 2.0 Hz, 1H, H-6), 7.15 (d, *J* = 2.0 Hz, 1H, H-8), 7.50 (d, *J* = 8.7 Hz, 1H, H-5), 9.19 (s, 1H, N-H). ^13^C-NMR (75 MHz, CDCl_3_): δ = 12.0 (*C*H_3_-CH_2_), 18.7 (CH_3_-4), 26.0 (O=C-*C*H_3_), 47.8 (C-2'/C-6'), 53.3 (C-3'/C-5'), 52.5 (N-*C*H_2_), 101.4 (C-8), 110.7 (C-6), 112.0 (C-3), 114.5 (C-4a), 125.9 (C-5), 145.0 (C-4), 145.8 (C-7), 152.5 (-C=N), 155.3 (C-8a), 161.3 (C-2), 195.0 (O=*C*-Me). Anal. Calcd for C_19_H_24_N_4_O_3_ (356.42 g/mol): C, 64.03; H, 6.79; N, 15.72. Found: C, 63.86; H, 6.68; N, 15.54. ESI-HRMS *m/z*: Calcd for C_19_H_25_N_4_O_3_ [M + H]^+^ 357.19267. Found: 357.19212.

*4-Methyl-7-{2-[2-oxo-1-(4-propylpiperazin-1-yl)propylidene]hydrazinyl}-2H-chromen-2-one* (**3g**): Yield = 0.51g (76.5%); Mp = 178-181 °C. ^1^H-NMR (300 MHz, CDCl_3_): δ = 0.91 (t, *J* = 7.2 Hz, 3H, C*H*_3_CH_2_-), 1.53 (m, 2H, MeC*H*_2_-), 2.39 (d, *J* = 1.0 Hz, 3H, C*H*_3_-4), 2.41 (s, 3H, O=C-C*H*_3_), 2.48 (t, *J* = 7.2, 2H, N-C*H*_2_-), 2.56 (m, 4H, H_2_-3' + H_2_-5' ), 3.09 (m, 4H, H_2_-2' + H_2_-6' ), 6.11 (d, *J* = 1.0 Hz, 1H, H-3), 7.04 (dd, *J* = 8.7 Hz, 2.0 Hz, 1H, H-6), 7.15 (d, *J* = 2.0 Hz, 1H, H-8), 7.50 (d, *J* = 8.7 Hz, 1H, H-5), 9.20 (s, 1H, N-H). ^13^C-NMR (75 MHz, CDCl_3_): δ = 12.0 (*C*H_3_CH_2_-), 18.8 (CH_3_-4), 20.0 (Me*C*H_2_-), 26.0 (O=C-*C*H_3_), 47.9 (C-2'/C-6'), 53.8 (C-3'/C-5'), 60.7 (N-*C*H_2_), 101.4 (C-8), 110.7 (C-6), 112.0 (C-3), 114.5 (C-4a), 125.8 (C-5), 145.0 (C-4), 145.8 (C-7), 152.6 (-C=N), 155.3 (C-8a), 161.4 (C-2), 195.2 (O=*C*-Me). Anal. Calcd. for C_20_H_26_N_4_O_3_ (370.45 g/mol): C, 64.84; H, 7.07; N, 15.12. Found: C, 64.63; H, 6.92; N, 15.05. ESI-HRMS *m/z*: Calcd. for C_20_H_27_N_4_O_3 _[M + H]^+^ 371.20832. Found: 371.20793.

*7-{2-[1-(4-Benzylpiperazin-1-yl)-2-oxopropylidene]hydrazinyl}-4-methyl-2H-chromen-2-one* (**3h**)*:* Yield = 0.46 g (61.1%); Mp = 199-201 °C. ^1^H-NMR (300 MHz, CDCl_3_): δ = 2.39 (d, *J* = 1.0 Hz, 3H, C*H*_3_-4), 2.41 (s, 3H, O=C-C*H*_3_), 2.55 (m, 4H, H_2_-3' + H_2_-5' ), 3.07 (m, 4H, H_2_-2' + H_2_-6' ), 3.57 (s, 2H, N-C*H*_2_-), 6.12 (d, *J* = 1.0 Hz, 1H, H-3), 7.04 (dd, *J* = 8.7 Hz, 2.0 Hz, 1H, H-6), 7.15 (d, *J* = 2.0 Hz, 1H, H-8), 7.26-7.33 (m, 5H, H-2''/H-3''/H-4''/H-5''/H-6''), 7.50 (d, *J* = 8.7 Hz, 1H, H-5), 9.19 (s, 1H, N-H). ^13^C-NMR (75 MHz, CDCl_3_): δ = 18.7 (CH_3_-4), 26.0 (O=C-*C*H_3_), 48.0 (C-2'/C-6'), 53.7 (C-3'/C-5'), 63.2 (N-*C*H_2_), 101.4 (C-8), 110.7 (C-6), 112.1 (C-3), 114.5 (C-4a), 125.9 (C-5), 127.2 (C-4''), 128.3 (C-2''/C-6''), 129.1 (C-3''/C-5''), 137.9 (C-1''), 145.2 (C-4), 145.8 (C-7), 152.3 (-C=N), 155.3 (C-8a), 161.3 (C-2), 195.1 (O=*C*-Me). Anal. Calcd. for C_24_H_26_N_4_O_3_ (418.49 g/mol): C, 68.88; H, 6.26; N, 13.39. Found: C, 68.96; H, 6.18; N, 13.24. ESI-HRMS *m/z*: Calcd. for C_24_H_25_N_4_O_3 _[M − H]^-^ 417.19267. Found: 417.19212.

*Ethyl 4-{1-[2-(4-methyl-2-oxo-2H-chromen-7-yl)hydrazono]-2-oxopropyl}piperazine-1-carboxylate* (**3i**): Yield = 0.52 g (72.2%); Mp = 221-223 °C. ^1^H-NMR (300 MHz, CDCl_3_): δ = 1.26 (t, *J* = 7.1 Hz, 3H, C*H*_3_-CH_2_), 2.39 (d, *J* = 1.0 Hz, 3H, C*H*_3_-4), 2.42 (s, 3H, O=C-C*H*_3_), 3.02 (m, 4H, H_2_-3' + H_2_-5' ), 3.58 (m, 4H, H_2_-2' + H_2_-6' ), 4.15 (q, *J* = 7.1 Hz, 2H, MeC*H*_2_ ), 6.13 (d, *J* = 1.0 Hz, 1H, H-3), 7.05 (dd, *J* = 8.7 Hz, 2.0 Hz, 1H, H-6), 7.19 (d, *J* = 2.0 Hz, 1H, H-8), 7.50 (d, *J* = 8.7 Hz, 1H, H-5), 9.27 (s, 1H, N-H). ^13^C-NMR (75 MHz, CDCl_3_): δ = 14.7 (*C*H_3_CH_2_-), 18.7 (CH_3_-4), 25.9 (O=C-*C*H_3_), 44.3 (C-2'/C-6'), 47.9 (C-3'/C-5'), 61.7 (Me*C*H_2_-), 101.6 (C-8), 110.8 (C-6), 112.3 (C-3), 114.7 (C-4a), 125.9 (C-5), 144.5 (C-4), 145.5 (C-7), 152.5 (-C=N), 155.3 (C-8a), 155.5 (O=*C*-N), 161.2 (C-2), 195.1 (O=*C*-Me). Anal. Calcd. for C_20_H_24_N_4_O_5_ (400.43 g/mol): C, 59.99; H, 6.04; N, 13.99. Found: C, 60.12; H, 6.01; N, 13.78. ESI-HRMS *m/z*: Calcd. for C_20_H_23_N_4_O_5 _[M − H]^-^ 399.16684. Found 399.16630.

*7-{2-[1-(4-(2-Hydroxyethyl)piperazin-1-yl)-2-oxopropylidene]hydrazinyl}-4-methyl-2H-chromen-2-one* (**3j**): Yield = 0.46 g (68.1%); Mp = 159-162 °C. ^1^H-NMR (300 MHz, CDCl_3_): δ = 2.39 (d, *J* = 1.0 Hz, 3H, C*H*_3_-4), 2.42 (s, 3H, O=C-C*H*_3_), 2.61 (t, *J* = 5.2 Hz, 2H, -NC*H*_2_CH_2_OH), 2.76 (m, 4H, H_2_-3' + H_2_-5'), 3.09 (m, 4H, H_2_-2' + H_2_-6'), 3.63 (t, *J* = 5.2 Hz, 2H, -NCH_2_C*H*_2_OH), 6.13 (d, *J* = 1.0 Hz, 1H, H-3), 7.05 (dd, *J* = 8.7, 2.0 Hz, 1H, H-6), 7.15 (d, *J* = 2.0 Hz, 1H, H-8), 7.51 (d, *J* = 8.7 Hz, 1H, H-5), 9.20 (s, 1H, N-H). ^13^C-NMR (75 MHz, CDCl_3_): δ = 18.7 (*C*H_3_-4), 25.9 (O=C-*C*H_3_), 48.0 (C-2'/C-6'), 53.5 (C-3'/C-5'), 57.7 (-N*C*H_2_CH_2_OH), 59.5 (-NCH_2_*C*H_2_OH), 101.6 (C-8), 110.7 (C-6), 112.2 (C-3), 114.6 (C-4a), 125.9 (C-5), 144.9 (C-4), 145.7 (C-7), 152.5 (-C=N), 155.3 (C-8a), 161.3 (C-2), 195.2 (O=*C*-Me). Anal. Calcd. for C_19_H_24_N_4_O_4 _(372.42 g/mol): C, 61.28; H, 6.50; N, 15.04. Found: C, 61.02; H, 6.41; N, 14.88. ESI-HRMS *m/z*: Calcd. for C_19_H_25_N_4_O_4 _[M + H]^+^ 373.18758. Found: 373.18707.

*7-{2-[1-(4-(2-Fluorophenyl)piperazin-1-yl) -2-oxopropylidene]hydrazinyl}-4-methyl-2H-chromen-2-one* (**3k**): Yield = 0.42 g (55.3%); Mp = 256-258 °C. ^1^H-NMR (300 MHz, CDCl_3_): δ = 2.36 (d, *J* = 1.0 Hz, 3H, C*H*_3_-4), 2.44 (s, 3H, O=C-C*H*_3_), 3.21 (m, 4H, H_2_-3' + H_2_-5' ), 3.24 (m, 4H, H_2_-2' + H_2_-6' ), 6.13 (d, *J* = 1.0 Hz, 1H, H-3), 6.95-7.00 (m, 4H, H-3'' + H-4'' + H-5'' + H-6''), 7.05 (dd, *J* = 8.7 Hz, 2.1 Hz, 1H, H-6), 7.18 (d, *J* = 2.1 Hz, 1H, H-8), 7.51 (d, *J* = 8.7 Hz, 1H, H-5), 9.25 (s, 1H, N-H). ^13^C-NMR (75 MHz, CDCl_3_): δ = 18.7 (CH_3_-4), 26.0 (O=C-*C*H_3_), 48.2 (C-2'/C-6'), 51.3 (d, *J* = 3.2 Hz, C-3'/ C-5'), 101.5 (C-8), 110.7 (C-6), 112.2 (C-3), 114.6 (C-4a), 116.3 (d, ^2^*J*_C-F_ = 20.6 Hz, C-3''), 119.2 (d, ^4^*J*_C-F_ = 2.7 Hz, C-5''), 122.9 (d, ^3^*J*_C-F_ = 7.9 Hz, C-4''), 124.4 (d, ^3^*J*_C-F_ = 3.5 Hz, C-6''), 125.9 (C-5), 139.5 (d, ^2^*J*_C-F_ = 19.7 Hz, C-1''), 144.9 (C-4), 145.7 (C-7), 152.4 (-C=N), 155.3 (C-8a), 155.9 (d, ^1^*J*_C-F_ = 245 Hz, C-2''), 161.2 (C-2), 195.1 (O=*C*-Me). Anal. Calcd. for C_23_H_23_FN_4_O_3_ (422.45 g/mol): C, 65.39; H, 5.49; N, 13.26. Found: C, 65.18; H, 5.40; N, 13.15. ESI-HRMS *m/z*: Calcd. for C_23_H_22_FN_4_O_3 _[M − H]^-^ 421.16759. Found: 421.16814.

*7-{2-[1-(4-(4-Fluorophenyl)piperazin-1-yl)-2-oxopropylidene]hydrazinyl}-4-methyl-2H-chromen-2-one* (**3l**): Yield = 0.45 g (59.3%); Mp = 271-273 °C. ^1^H-NMR (300 MHz, CDCl_3_): δ = 2.40 (d, *J* = 1.0 Hz, 3H, C*H*_3_-4), 2.45 (s, 3H, O=C-C*H*_3_), 3.21 (m, 4H, H_2_-3' + H_2_-5' ), 3.22 (m, 4H, H_2_-2' + H_2_-6' ), 6.14 (d, *J* = 1.0 Hz, 1H, H-3), 6.89-6.98 (m, 4H, H-2'' + H-3'' + H-5'' + H-6''), 7.03 (dd, *J* = 8.7 Hz, 2.1 Hz, 1H, H-6), 7.18 (d, *J* = 2.1 Hz, 1H, H-8), 7.51 (d, *J* = 8.7 Hz, 1H, H-5), 9.24 (s, 1H, N-H). ^13^C-NMR (75 MHz, CDCl_3_): δ = 18.7 (CH_3_-4), 26.0 (O=C-*C*H_3_), 48.2 (C-2'/C-6'), 51.3 (C-3'/C-5'), 101.6 (C-8), 110.7 (C-6), 112.2 (C-3), 114.7 (C-4a), 115.7 (d, ^2^*J*_C-F_ = 22 Hz, C-3''/C-5''), 118.3 (d, ^3^*J*_C-F_ = 7.7 Hz, C-2''/C-6''), 157.2 (d, ^1^*J*_C-F_ = 240 Hz, C-4''), 125.9 (C-5), 147.3 (d, ^4^*J*_C-F_ = 2.2 Hz, C-1''), 144.8 (C-4), 145.6 (C-7), 152.5 (-C=N), 155.3 (C-8a), 161.2 (C-2), 195.2 (O=*C*-Me). Anal. Calcd. for C_23_H_23_FN_4_O_3_ (422.45 g/mol): C, 65.39; H, 5.49; N, 13.26. Found: C, 65.22; H, 5.45; N, 13.18. ESI-HRMS *m/z*: Calcd. for C_23_H_24_FN_4_O_3 _[M + H]^+^ 423.18324. Found: 423.18270.

*4-Methyl-7-(2-(2-oxo-1-(4-(pyrimidin-2-yl)piperazin-1-yl)propylidene)hydrazinyl)-2H-chromen-2-one* (**3m**): Yield = 0.55 g (75.2%); Mp = 236-238 °C. ^1^H-NMR (300 MHz, CDCl_3_): δ = 2.39 (d, *J* = 1.0 Hz, 3H, C*H*_3_-4), 2.43 (s, 3H, O=C-C*H*_3_), 3.12 (m, 4H, H_2_-3' + H_2_-5' ), 3.94 (m, 4H, H_2_-2' + H_2_-6' ), 6.12 (d, *J* = 1.0 Hz, 1H, H-3), 6.52 (t, *J* = 4.8 Hz, 1H, H-5''), 7.06 (dd, *J* = 8.7, 2.0 Hz, 1H, H-6), 7.20 (d, *J* = 2.0 Hz, 1H, H-8), 7.52 (d, *J* = 8.7 Hz, 1H, H-5), 8.32 (d, *J* = 4.8 Hz, 2H, H-4'' + H-6''), 9.35 (s, 1H, N-H). ^13^C-NMR (75 MHz, CDCl_3_): δ = 18.7 (CH_3_-4), 26.0 (O=C-*C*H_3_), 44.4 (C-2'/C-6'), 47.9 (C-3'/C-5'), 101.6 (C-8), 110.3 (C-5''), 110.8 (C-6), 112.2 (C-3), 114.7 (C-4a), 125.9 (C-5), 144.8 (C-4), 145.7 (C-7), 152.5 (-C=N), 155.3 (C-8a), 157.8 (C-4'' + C-6''), 159.8 (C-2''), 161.2 (C-2), 195.2 (O=*C*-Me). Anal. Calcd. for C_21_H_22_N_6_O_3_ (406.44 g/mol): C, 62.06; H, 5.46; N, 20.68. Found: C, 61.87; H, 5.38; N, 20.52. ESI-HRMS *m/z*: Calcd. for C_21_H_21_N_6_O_3 _[M − H]^-^ 405.16751. Found: 405.16696.

*7-{2-[1-(4-(1-benzyl-2-ethyl-4-nitro-1H-imidazol-5-yl)piperazin-1-yl)-2-oxopropylidene]hydrazinyl}-4-methyl-2H-chromen-2-one* (**3n**): Yield = 0.89 g (88.7%); Mp = 193-195 °C. ^1^H-NMR (300 MHz, CDCl_3_): δ = 1.28 (t, *J* = 7.5 Hz, 3H, CH_2_-C*H*_3_), 2.39 (d, *J* = 1.0 Hz, 3H, C*H*_3_-4), 2.41 (s, 3H, O=C-C*H*_3_), 2.61 (q, *J* = 7.5 Hz, 2H, C*H*_2_-Me), 2.68 (m, 4H, H_2_-3' + H_2_-5' ), 3.36 (m, 4H, H_2_-2' + H_2_-6' ), 5.17 (s, 2H, C*H*_2_-Ph), 6.12 (d, *J* = 1.0 Hz, 1H, H-3), 7.00 (dd, *J* = 8.7 Hz, 2.0 Hz, 1H, H-6), 7.02 (d, *J* = 7.5 Hz, 2H, H-2'''/ H-6'''), 7.30 (d, *J* = 2.0 Hz, 1H, H-8), 7.32-7.37 (m, 3H, H-3''' + H-4''' + H-5'''), 7.51 (d, *J* = 8.7 Hz, 1H, H-5), 9.34 (s, 1H, N-H). ^13^C-NMR (75 MHz, CDCl_3_): δ = 11.3 (-CH_2_-*C*H_3_), 18.8 (CH_3_-4), 21.2 (-*C*H_2_-Me), 25.9 (O=C-*C*H_3_), 46.2 (*C*H_2_-Ph), 48.4 (C-2'/C-6'), 49.6 (C-3'/C-5'), 101.6 (C-8), 111.1 (C-6), 112.2 (C-3), 114.8 (C-4a), 125.9 (C-5), 126.0 (C-2'''/C-6'''), 128.3 (C-4'''), 129.3 (C-3'''/C-5'''), 135.4 (C-1'''), 139.1 (C-1''), 140.0 (C-5''), 144.5 (C-4), 145.3 (C-4''), 145.5 (C-7), 152.5 (Ac-*C*=N), 155.3 (C-8a), 161.3 (C-2), 195.4 (O=*C*-Me). Anal. Calcd. for C_29_H_31_N_7_O_5_ (557.60 g/mol): C, 62.47; H, 5.60; N, 17.58. Found: C, 62.54; H, 5.64; N, 17.42. ESI-HRMS *m/z*: Calcd. for C_29_H_31_N_7_NaO_5 _[M + Na]^+^ 580.22844. Found: 580.22789.

### 3.5. Cell Lines and Cell Culture

#### Materials and Methods

Human breast cancer cell line MCF-7, was a gift from Drs. Prakash and Mitzi Nagarkatti (University of South Carolina, School of Medicine, Columbia, SC, USA). Human leukemia HL-60-Acute Myelocytic Leukemia (AML) and K5626 were gifts from Salem Akel at Hashemite University, Zarqa, Jordan. The three cell lines were maintained in complete RPMI-1640 medium supplemented with 10% heat-inactivated fetal bovine serum, 2 mM L-glutamine, 100 units/mL penicillin, 100 μg/mL streptomycin (all from Gibco), 50 μM 2-mercaptoethanol (Research Organics, USA), 10 mM HEPES buffer (AppliChem, Germany), and gentamicin sulfate at 0.05 mg/mL. Cells were maintained under standard culture conditions at 37 °C in a water-saturated atmosphere of 5% CO_2_ in air. ZR-75-1 breast cancer cells (obtained from ATCC) were cultured in DMEM supplemented with 2 mM glutamine and 10% Fetal Bovine Serum (FBS, Gibco Life Technologies). 

#### 3.2.2 Cell Proliferation Assay

MCF-7 and K562 cells were seeded at a density of 1 × 10^4^ and 4 × 10^4^ per well in 96-well plates in appropriate medium, then treated with 50 μM concentrations of the tested compounds. For the IC_50_ determination, the cells were treated with increasing concentrations of the tested compound (1.56-100 μM). In all assays, the drugs were dissolved in DMSO immediately before the addition to cell cultures and equal amounts of the solvent were added to control cells; the final concentration of DMSO did not exceed 1%. Cell viability was assessed, after 3 days of treatment, with tetrazolium dye 3-(4,5-dimethylthiazol-2-yl)-2,5-diphenyltetrazolium bromide (MTT), obtained from Sigma (Dorset, UK). IC_50_ concentrations were obtained from the dose-response curves using Graph Pad Prism Software 5 (San Diego, CA, USA, www.graphpad.com), and doxorubicin as positive control. 
